# Editorial: The Application of Proteogenomics to Urine Analysis for the Identification of Novel Biomarkers of Prostate Cancer: An Exploratory Study

**DOI:** 10.3390/cancers15164143

**Published:** 2023-08-17

**Authors:** Yaser Gamallat, Tarek A. Bismar

**Affiliations:** 1 Department of Pathology and Laboratory Medicine, Arnie Charbonneau Cancer Institute, Cumming School of Medicine, University of Calgary, Calgary, AB T2N 4N1, Canada; yaser.gamallat@ucalgary.ca; 2 Department of Oncology, Biochemistry and Molecular Biology, Arnie Charbonneau Cancer Institute, Cumming School of Medicine, University of Calgary, Calgary, AB T2N 4N1, Canada; 3 Departments of Pathology & Laboratory Medicine, Alberta Precision Laboratories, Rockyview General Hospital, Calgary, AB T2V 1P9, Canada; 4 Prostate Cancer Center, Rockyview General Hospital, Calgary, AB T2V 1P9, Canada

**Keywords:** proteogenomics, prostate cancer, biomarker detection

## Abstract

In this editorial context, we aim to leverage the potential of proteogenomics, which integrates genomic and proteomic data, to discover novel biomarkers that can aid in the diagnosis and management of prostate cancer. We highlight the importance of proteogenomics for understanding the functional consequences of somatic mutations in cancer and demonstrating how proteogenomic analysis can provide insights into the effects of genetic alterations on the proteomic landscape and identify potential therapeutic targets. This article also emphasizes the potential of urine analysis for the detection of prostate cancer. Overall, our editorial paper provides general insights on the application of proteogenomics to urine analysis for the identification of novel biomarkers of prostate cancer.

Prostate cancer (PCa) is the most common cancer in men in the United States, and it is the second most common cancer among men worldwide [1]. The early detection of PCa is essential for successful treatment and improved patient outcomes. In recent years, researchers have been increasingly challenged to develop innovative tools able to detect cancer during its early stages, when it is the most curable. The aim is to identify tumor-specific compounds using biological fluids. The transformation and development of tumor cells possibly causes abnormal alterations leading to the release of cells, extracellular vesicles (EVs), circulating miRNAs, Long noncoding RNAs and metabolite compounds. In this editorial letter, we summarize some recent studies and approaches investigated as a discovery platform of urine proteogenomics towards the identification of novel biomarkers of prostate cancer (PCa). In the last decade, many studies have focused on the identification of cancer-characteristic fingerprints from biological samples through the application of sensorial or senso-instrumental analyses, while others have suggested a chemical characterization of biological fluids with the aim of identifying prostate-cancer (PCa)-specific biomarkers. However, recent articles have investigated the application of proteogenomics to identify novel and reliable diagnostic biomarkers based on urine analysis, which seems to be of special interest as a new direction for biomarker discovery [2].

Lima et al. [3] used a combination of mass spectrometry and genomic data to identify proteins and genomic mutations that were co-expressed in urine samples from patients with prostate cancer compared with healthy controls. They identified several potential biomarkers that could be used for the early detection of PCa.

The significance of this study is that it provides a newer and easier approach to some of the current challenges for identifying prostate cancer biomarkers through urine analysis. This approach could be used to develop new diagnostic tests for prostate cancer that are less invasive than current methods [4,5,6].

Currently, the most common screening biomarker for PCa diagnosis and monitoring is prostate-specific antigen (PSA) serum level in association with a digital rectal exam (DRE) [7,8,9,10,11,12,13]. PSA is serine protease secreted into the fluid of the glandular ducts and is responsible for the controlled release of sperm. In a normal prostate, only small amounts of PSA (i.e., <4 ng/mL) present in the extracellular fluid and diffuse into circulation. However, in PCa, higher serum level of PSA usually occur due to the disorganizing and polarization of the epithelial cells’ architecture, which causes a loss of normal secretory functions into the prostatic ducts. Nonetheless, PSA tests have limitations, including a lack of specificity and sensitivity, (i.e., a specificity around 33% and a sensitivity around 86%) leading to overdiagnosis and overtreatment.

Proteomics and genomics are two powerful technologies that can provide new insights into the molecular mechanisms of diseases, including PCa. Proteomics involves the large-scale study of proteins, while genomics focuses on the complete set of genes and their functions. Proteogenomics is a relatively new field that combines proteomics and genomics to study the relationship between genes and proteins. The integration of proteomic and genomic data can provide a more comprehensive understanding of disease mechanisms and lead to the identification of novel biomarkers.

The authors enrolled 35 patients with PCa and 35 healthy controls. Five cases with PCa and five controls were recruited as discovery controls, and the remaining thirty cases from both groups were used as testing controls.

Initially, the authors performed MS proteomics on the urine collected from the PCa patients and healthy controls. The data collected were analyzed using two different software packages to boost the results using multiple resources. Only the top-ranking protein of each group was considered, and about 732 proteins in total were identified. The authors performed Principal Component Analysis and a heatmap of the identified top proteins and obtained two protein cluster discriminations using max quant data that separated clusters into proteins upregulated in PCa patients compared with non-cancer subjects, while the second cluster proteins were predominantly downregulated in PCa patients.

Altogether, the authors were identified 18 dysregulated proteins in PCa samples by using differentially expressed protein analyses in which 11 proteins were significantly downregulated (with fold change < 1) and 7 proteins were significantly upregulated (fold change > 1).

Five protein targets were identified by the two software packages: Cadherin-1 (CDH1), EGF-containing fibulin-like extracellular matrix protein 1 (EFEMP1), Prostate-specific antigen (PSA) (KLK3), Secreted and transmembrane protein 1 (SECTM1) and Transthyretin (TTR). Interestingly, the most widely known biomarker for PCa clinical diagnosis, PSA, was one of the dysregulated proteins in common in the analysis by both software packages. Overall, the authors remarkably found decreased levels of proteins SECTM1, CDH13, AMY2A, EFEMP1, ITIH4, HSPG2, PTGDS, CDH1 and LMAN2 and increased levels of TTR and KLK3 protein expression in the urine proteomes of PCa patients.

The authors also performed immunoblot tests to confirm the reproducibility of the MS data for the five selected protein targets, AMBP, CDH1, EFEMP1, LMAN2 and TTR. However, none of the MS findings could be reproduced; even the urinary PSA levels in the testing cohort did not agree with the MS findings.

The authors then looked at the proteogenomic mutations landscape of urine samples from PCa, which revealed 6418 urinary mutated peptides. The authors selected the high-confidence proteins associated with prostate cancer, resulting in 86 proteins with known expression in PCa, like Acid ceramidase (ASAH1), Glutathione S-transferase P (GSTP1), Extracellular superoxide dismutase [Cu-Zn] (SOD3), Osteopontin (SPP1), Prostatic acid phosphatase (PAP) and Zinc-alpha-2-glycoprotein (ZAG).

The authors attempted to explore the urine proteogenomic data. Interestingly, they found that no group separation was observed in the Principal Component Analysis (PCA) of the proteogenomic profile of PCa patients, but heatmap analyses indicated a discrimination between PCa patients and healthy subjects based on two protein clusters: ITIH4*G893S (Inter-alpha-trypsin inhibitor heavy chain H4)-LMAN2*D222N (Vesicular integral-membrane protein VIP36) and KLK3*C209Y (PSA)-MVB12B*T198M (Multivesicular body subunit 12B). They concluded that, in the first cluster, mutant forms of proteins are mostly downregulated in PCa patients compared with healthy subjects, while in the second cluster, mutant forms of proteins are upregulated predominantly in PCa patients.

When they integrated the above data with the Cancer Genome Atlas (TCGA), DisGeNET and the available literature, the authors found only three of the mutations from the 86 proteins with known prostate expressions. These mutations (rs17632542, rs1695, rs7041) were mapped on KLK3 (PSA), GSTP1 (Glutathione S-transferase P) and GC (Vitamin D-binding protein). The remaining mutant protein isoforms with PCa had no mutation related to PCa described anywhere in the literature up to that date.

Again, the authors looked back at the level difference between the native and mutant forms of proteins in the urine from PCa patients. The proteogenomic data analyses revealed six abundant mutant protein isoforms in PCa urine samples, i.e., Protein AMBP (AMBP*A286G), Sodium/hydrogen exchanger 9B1 (SLC9B1*N70S), Basement membrane-specific heparan sulfate proteoglycan core protein (HSPG2*Q1062H), Zinc finger protein 624 (ZNF624*S207F), Vasorin (VASN*R161Q) and Complement decay-accelerating factor (CD55*S162L). Mutant AMBP isoform (AMBP*A286G; HSPG2*Q1062H) was upregulated in PCa patients, while the remaining five were downregulated. Some of the native forms of VASN and CD55 proteins were not found to be dysregulated, but their mutant protein isoforms (VASN*R161Q; CD55*S162L) were in urine samples from PCa patients. However, the remaining common proteins present with mutations did not show different abundance in urine.

The authors also tried to predict or determine the potential impact of point mutations on protein function or the likely impact of a single residue using a PolyPhen-2 tool. Notably, it was predicted that AMBP*A286G and CD55*S162L mutant protein isoforms were predicted to be probably damaging, while SLC9B1*N70S, ZNF624*S207F, VASN*R161Q and HSPG2*Q1062H were predicted to be benign.

The highlighted study has some limitations. First, the test sample size was small, which limits the generalizability of the findings. Second, the validation cohort was relatively small to confirm the findings, as we see no reproducibility when they performed immunoblots. Third, the study did not investigate the clinical utility of the identified biomarkers, as immunoblot would not be recommended but ELISA or other methods that are easy to access and perform would. Despite these limitations, the study provides a new approach for identifying biomarkers for prostate cancer using urine samples. This approach could lead to the development of new diagnostic tests that are less invasive and more accurate than current methods.

In conclusion, the study provides a new approach for identifying biomarkers for prostate cancer using urine samples. This approach could lead to the development of new diagnostic tests that are less invasive and more accurate than current methods. The study also identified several potential biomarkers that could be used for the early detection of prostate cancer, which could lead to earlier diagnosis and better outcomes for patients.

Recently, Yazbek Hanna, M. et al. (2023) [14] proposed gene-transcript expression in the urine of 76 men, 40 with localized PCa and 36 non-cancer. Both cell and EV fractions were analyzed using Nanostring for 154 PCa-associated gene-probes, with 11 tissue-specific probes. Differential gene expression analysis found 57 gene-probes significantly more highly expressed in 100 ng of amplified cDNA products from the EV fraction, and 26 in cells (*p* < 0.05; edgeR). Using statistical analysis, the authors identified 11 gene-probes as useful in detecting PCa: 2 were useful in both fractions (PCA3, HOXC6), 5 in EVs alone (GJB1, RPS10, TMPRSS2:ERG, ERG_Exons_4-5, HPN) and 4 from cells (ERG_Exons_6-7, OR51E2, SPINK1, IMPDH2).

## Conclusions

Research on developing reliable biomarkers is still needed. Identifying such biomarkers can provide valuable information about disease progression, aggressiveness and treatment response. Researchers have been investigating the presence of specific biomarkers in urine for PCa diagnosis. Some have used genomic RNA-based biomarkers, such as non-coding RNAs, such as microRNAs (miRNAs) and long non-coding RNAs (lncRNAs), which have shown promise as urine-based biomarkers for PCa. Specific RNA signatures or alterations in the expression patterns of these molecules can be detected and used for the diagnosis, risk stratification and monitoring of PCa patients. Similarly, combining this with DNA-based biomarkers for urine-based detection of specific DNA alterations, such as gene mutations, deletions or methylation changes, has been explored for PCa diagnosis. By comparing the protein profiles of patients with PCa and those without, researchers have discovered potential protein biomarkers that could aid in diagnosis and prognosis. The proteogenomic data may not limited to metabolomic biomarkers, which involve the metabolic profile of biological samples, to identify specific metabolites associated with disease. In the authors’ opinion, using proteogenomic biomarkers, i.e., mass spectrometry-based proteomics combined with genomics, to identify and characterize the proteins present in urine samples from PCa patients holds promise, but in-depth further research and validation are required before they can be implemented in routine clinical practice. And more importantly, including large sample numbers involving diverse patient populations is necessary to establish the reliability, sensitivity and specificity of these biomarkers.
